# Synthesis of Sulfo-Sialic Acid Analogues: Potent Neuraminidase Inhibitors in Regards to Anomeric Functionality

**DOI:** 10.1038/s41598-017-07836-y

**Published:** 2017-08-15

**Authors:** Christopher J. Vavricka, Chiaki Muto, Tomohisa Hasunuma, Yoshinobu Kimura, Michihiro Araki, Yan Wu, George F. Gao, Hiroshi Ohrui, Minoru Izumi, Hiromasa Kiyota

**Affiliations:** 1 Graduate School of Environmental and Life Science, Okayama University, Okayama, 700-8530 Japan; 2 Graduate School of Science, Technology and Innovation, Kobe University, Kobe, 657-0013 Japan; 3 CAS Key Laboratory of Pathogenic Microbiology and Immunology, Institute of Microbiology, Chinese Academy of Sciences, Beijing, 100101 China; 4 Yokohama College of Pharmacy, Yokohama, 245-0066 Japan

## Abstract

The design, synthesis and application of *N*-acetylneuraminic acid-derived compounds bearing anomeric sulfo functional groups are described. These novel compounds, which we refer to as sulfo-sialic acid analogues, include 2-decarboxy-2-deoxy-2-sulfo-*N*-acetylneuraminic acid and its 4-deoxy-3,4-dehydrogenated pseudoglycal. While 2-decarboxy-2-deoxy-2-sulfo-*N*-acetylneuraminic acid contains no further modifications of the 2-deoxy-pyranose ring, it is still a more potent inhibitor of avian-origin H5N1 neuraminidase (NA) and drug-resistant His275Tyr NA as compared to the oxocarbenium ion transition state analogue 2,3-dehydro-2-deoxy-*N*-acetylneuraminic acid. The sulfo-sialic acid analogues described in this report are also more potent inhibitors of influenza NA (up to 40-fold) and bacterial NA (up to 8.5-fold) relative to the corresponding anomeric phosphonic acids. These results confirm that this novel anomeric sulfo modification offers great potential to improve the potency of next-generation NA inhibitors including covalent inhibitors.

## Introduction

Neuraminidase (NA, EC 3.2.1.18), also referred to as sialidase, is a retaining glycosidase that hydrolyzes α-ketosidic linkages to sialic acids. Common substrates recognized by NA contain terminal *N*-acetylneuraminic acid (Neu5Ac, NANA) or *N*-glycolylneuraminic acid (Neu5Gc, NGNA) with an α2-3 or α2-6 ketosidic bond to galactose. NA is found in many types of pathogens including *Trypanosoma cruzi*, *Clostridium perfringens*, *Streptococcus pneumoniae, Vibrio cholerae*, mumps virus, parainfluenza virus and influenza virus. By destroying sialic acid-containing glycosides, NA facilitates the movement of pathogens through biological environments rich in sialic acid receptors, including mammalian tissues and mucus. Therefore inhibition of NA can hinder the spread of sialic acid binding pathogens, and NA inhibitors offer great potential as anti-microbial agents. In the case of sialic acid binding viruses, NA also facilitates the release of progeny virions from infected cells. Accordingly, NA inhibitors have been especially successful as anti-influenza pharmaceuticals^1, 2^.

**Figure 1:**
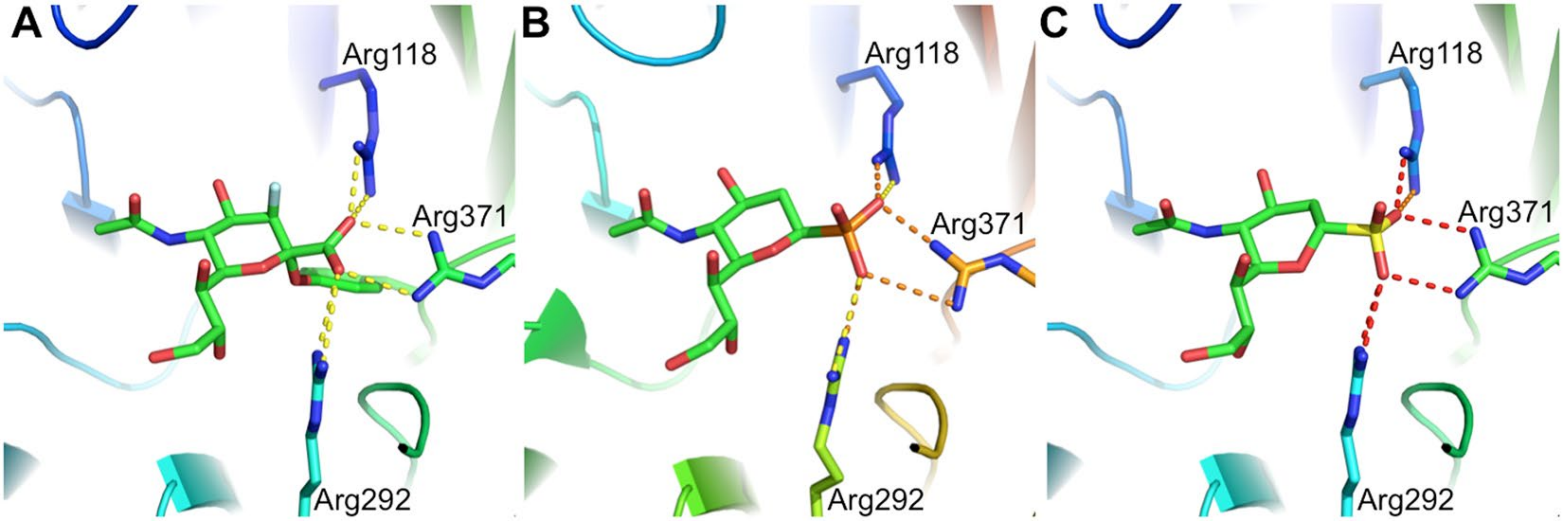
NA binding comparison of anomeric carboxy, anomeric phosphono and anomeric sulfo analogues of *N*-acetylneuraminic acid. (**A**) The carboxylate of 3-fluoro-Neu5Ac covalently bound to influenza A/RI/5+/1957(H2N2) NA (N2) NA (PDB ID: 45H2)^3^. (**B**) An equatorial phosphonate Neu5Ac analogue (eqPO_3_H) bound to influenza A/Tokyo/3/67(H2N2) NA with the phosphorus atom colored orange (PDB ID: 1INX)^6^. (**C**) Predicted binding of the equatorial sulfo-sialic acid analogue **7a** to N2 with the sulfur atom colored yellow (based on PDB ID: 45H2). The structure of **7a** was modeled in the N2 active site using Molecular Operating Environment (MOE). Electrostatic interactions with the anomeric functional groups are shown as dashed lines with stronger predicted ionic interactions colored orange and red.

The first generation of NA inhibitors was based upon Neu5Ac and its dehydrated product 2,3-dehydro-2-deoxy-*N*-acetylneuraminic acid (Neu5Ac2en or DANA). Currently, all clinically used NA inhibitors contain an anomeric carboxy group, which forms strong electrostatic interactions with three structurally conserved arginine residues in the NA active site (Fig. 1)^1^. Despite a lack of sequence identity among NAs from unrelated species, many active site structural features, including the triarginyl cluster, are highly conserved across all known NAs. For example, in the case of influenza A NA, a glycoside hydrolase family 34 member, this triarginyl cluster consists of Arg118, Arg292 and Arg371 (Fig. 1)^3^. In the case of *Clostridium perfringens* NanI NA, a glycoside hydrolase family 33 member, the arginine triad consists of Arg266, Arg555 and Arg615.

Replacement of the inhibitor carboxy group with a phosphono group has previously been reported to improve NA inhibitory activity and therefore phosphonic acid analogues of sialic acid are currently under development^4–10^. This increase in potency has been attributed to stronger electrostatic interactions of the anomeric phosphono with the conserved active site NA triarginyl cluster (Fig. 1)^5, 6^.

Based upon the higher acidity and electronegativity of sulfo relative to carboxy and phosphono functional groups, we predicted that sialic acid anomeric sulfonic acid analogues, which we refer to as sulfo-sialic acid analogues, should form the strongest electrostatic interactions with the conserved NA active site triarginyl cluster (Fig. 1). This strategy has been considered in an *in silico* analysis, which also indicated that the sulfo group should produce the strongest binding inhibitors^11^. Yet, the actual synthesis of sulfo-sialic acid analogues is quite challenging and has remained unreported. In fact, with exception to 2-amino and 2-acetamido anomeric sulfonates, no 2-deoxy sugar with an anomeric sulfo group has been reported to our knowledge^12, 13^.

We first became interested in the synthesis of sulfo-sialic acid analogues during our research on covalent NA inhibitors, which have utilized an electronegative 3-fluoro group to destabilize formation of oxocarbenium ion transition states (Fig. 1A)^3^. In addition to enhancing electrostatic interactions with the triarginyl cluster, a strong electron withdrawing sulfo group might also destabilize oxocarbenium ion formation. In this report we reveal the first synthesis of sulfo-sialic acid analogues and their application as potent NA inhibitors.

## Results and Discussion

**Figure 2:**
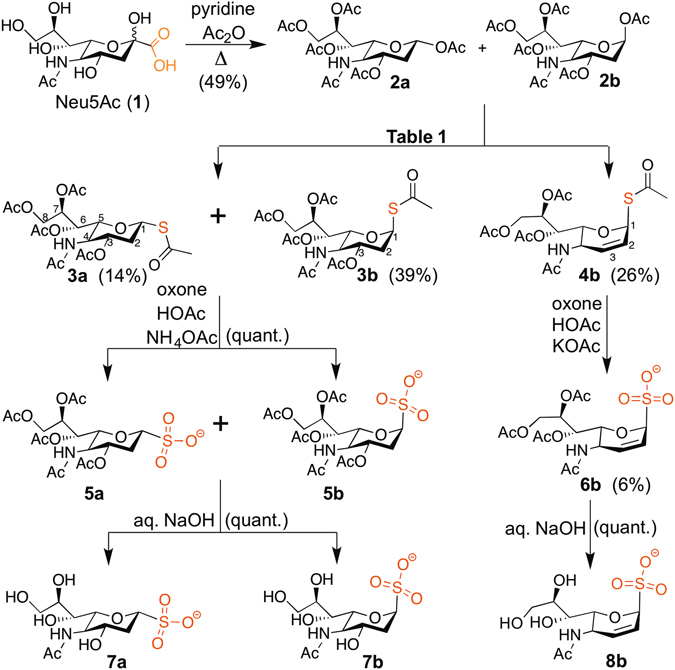
Synthesis of sulfo-sialic acid analogues. α-Anomers﻿﻿ **2a**, **3a**, **5a** and **7a** are in the *R* anomeric configuration with equatorial anomeric functional groups, and ﻿β﻿﻿-anomers **2b**, **3b**, **4b**, **5b**, **6b**, **7b** and **8b** are in the *S* anomeric configuration with axial anomeric functional groups.

Sulfo-sialic acid analogues were synthesized via oxidation of a mixture of acetylthio intermediates according to Fig. 2. Neu5Ac (**1**)-derived peracetylated octoses **2a** and **2b**, first reported by Potter and von Itzstein, were selected as the substrate for sulfur addition^14^. The decarboxylated Neu5Ac derivatives **2a** and **2b** were synthesized based on the method of Shie *et al*. with modifications^5^.

Nucleophilic substitution of the **2** anomeric position with HSAc was mediated by either BF_3_·Et_2_O or TMSOTf, using CH_3_CN, CHCl_3_, or 1,2-dichloroethane ﻿(1,2-DCE)﻿ as solvent (Table 1). Although our original intention was to produce only **3**, during NMR analysis of the acetylthio reaction products, a surprisingly large peak was always observed at 5.80 ppm, with coupling to another singlet at 6.27 ppm. These NMR peaks were later attributed to H-1, H-2 and H-3 of the 2,3-unsaturated pseudoglycal **4b**, similar to compounds resulting from the Ferrier rearrangement of glycals in which the axial orientation of the anomeric acetylthio group is also preferred^15, 16^. The axial orientation of the acetylthio group of **4b** is indicated by a lack of NOE correlation between H-1 and H-5. The structure of **4b** was further supported by high-resolution mass spectrometry (HRMS), and characterization of the oxidation products **6b** and **8b**. The dominance of the axial acetylthio orientation of this pseudoglycal could be explained by the anomeric effect. Less polar reaction conditions were used to produce a higher ratio of **4b** to **3** (Table 1). More polar reaction conditions could be used to weaken the anomeric effect and increase the proportion of equatorial acetylthio compound **3a** (Table 1).

**Table 1:** Reaction conditions and yields for synthesis of acetylthio glycoside **3** and acetylthio pseudo-glycal **4b**.

2	Lewis Acid	HSAc	Solv.	Time	Temp.	Yield	3a:3b:4b
426 µmol, 115 mM	TMSOTf, 290 mM, 2.5 eq.	737 mM, 6.4 eq.	CH_3_CN	19 h.	−20 °C - r.t.	44%	33:36:31
1.97 mmol, 94 mM	BF_3_·Et_2_O, 684 mM, 7.2 eq.	746 mM, 7.9 eq.	1,2-DCE	45 h.	0 °C - r.t.	71%	17:54:29
673 µmol, 22 mM	BF_3_·Et_2_O, 53 mM, 2.4 eq.	56 mM, 2.5 eq.	1,2-DCE	11 h.	0 °C - r.t.	40%	6:28:66

Oxidation of the acetylthio functionality of **3** and **4** to the sulfo functionality was achieved using excess Oxone (potassium peroxymonosulfate, KHSO_5_﻿· 0.5KHSO_4_· 0.5K_2_SO_4_) in AcOH buffered with KOAc, based upon the method of Reddie^17–19^. Workup was somewhat difficult due to the presence of excess salt, so the procedure was modified with NH_4_OAc in place of KOAc for the oxidation of **3** to **5**, which appeared to be quantitative. Yields for the oxidation of **4** to **6** were low due to concurrent oxidation of the pseudoglycal ring double bond by the excess Oxone. During HRMS analysis of polar Oxone oxidation products, negative ions *m/z* 452.0826 (**6** + O), *m/z* 454.0985 (**6** + O + 2H) and *m/z* 470.0949 (**6** + 2O + 2H) were detected, indicating that **6** was further oxidized. Sulfonates **5a** and **5b** could also be converted to the corresponding methyl esters by reaction with trimethylsilyldiazomethane, a toxic reagent that should be handled with caution. Active sulfo-sialic acid analogues, **7a**, **7b** and **8b** were obtained after deprotection of the precursors in the presence of NaOH.

The structures of sulfo-sialic acid analogues **5-8** were determined using HRMS,^1^H, GCOSY, NOESY and^13^C data. H-1, H-2 and H-5 are all shifted downfield in the axial anomeric sulfonates **5b** and **7b** relative to the equatorial isomers **5a** and **7a**, which is also observed in **2**, **3**, and the phosphonic acid analogues of **5** and **7** ^4, 5^. The H-1 to H-2 coupling constants of **5a**, **5b**, **7a** and **7b** match those of the corresponding phosphonic acids and carboxylic acids as well^4, 5, 20^. A clear H-1 to H-5 NOE correlation is observed in the equatorial sulfo compound **7a**. In contrast, the lack of any NOE correlation between H-1 and H-5 in **8b** is consistent with the axial anomeric functional group orientations of **4b**, **6b** and **8b**. A clear H-1 to H-2 NOE correlation in **8b** further supports the equatorial orientation of H-1. Synthesis of psuedoglycals with anomeric sulfones has also been reported with axial anomeric stereoselectivity^21^. Isomerization of the anomeric position was never observed for any of the sulfo-sialic acid analogues.

Due to the high electronegativity of the sulfo group, the diagnostic H-1, H-2, H-5 and C-1 chemical shifts of **5** and **7** are shifted downfield relative to the corresponding carboxylic acids and phosphonic acids (Table 2). This observance of a strong electron-withdrawing inductive effect of the anomeric sulfo group supports our hypothesis that sulfo-sialic acid analogues will be useful for the development of next-generation covalent NA inhibitors^3^.

**Table 2:** Diagnostic ^13^C and ^1^H chemical shifts (ppm) of sulfo-sialic acid analogues compared to the corresponding phosphonic acids^4^, and carboxylic acids^20^.

	C-1 Anomeric	C-2	H-1 Anomeric	H-2ax	H-2eq	H-5	Solvent
**7a**	87.0	36.4	4.28	1.79	2.45	3.63	CD_3_OD
**7b**	86.7	34.3	4.70	1.92	2.62	4.25	CD_3_OD
eqPO_3_H	73.6	36.2	3.75	1.70	2.24	3.49	CD_3_OD
axPO_3_H	71.9	34.4	4.25	1.94	2.36	3.93	CD_3_OD
eqCO_2_H*	76.8	37.8	3.87	1.56	2.36	3.58	D_2_O
axCO_2_H*	74.8	35.2	4.44	1.87	2.56	3.62	D_2_O

*Sialic acid numbering has been changed to assign the anomeric position as 1.

**Figure 3:**
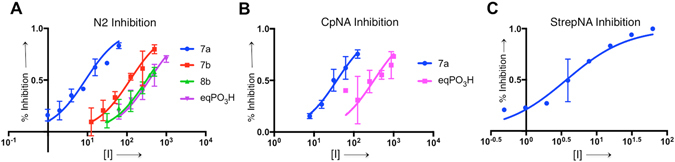
Sulfo-sialic acid analogue NA inhibition. Inhibition of N2 (**A**), CpNA (**B**) and StrepNA (**C**) hydrolysis of 4-methylumbelliferyl-Neu5Ac with **7a** (blue), **7b** (red), **8b** (green) and eqPO_3_H (magenta). Error bars indicate standard error ﻿and inhibitor concentrations (﻿x-axis) units are μ﻿M.

In order to assess the relative effectiveness of sulfo-sialic acid analogues, the anomeric phosphonic acids corresponding to **7a** (eqPO_3_H, ePANA) and **7b** (axPO_3_H, aPANA) were synthesized based upon the methods of Shie *et al*. with modifications^5^. Inhibition of influenza A/Anhui/1/2005 (H5N1) NA (N1), A/RI/5+/1957(H2N2) NA (N2), *Clostridium perfringens* NanJ NA (CpNA) and *Streptococcus* 6646K NA (StrepNA), was quantified using a fluorogenic assay based upon the method by Potier *et al*. with modifications^22^. Inhibition of influenza and bacterial NA by sulfo-sialic acid analogues confirms that the sulfo group enables more potent NA inhibition relative to the analogous phosphonic acids (Table 3, Fig. 3). The NA inhibitory activity of axPO_3_H was difficult to quantify due to its low potency. For inhibition of influenza N2, the equatorial anomeric sulfonate **7a** was 40-fold more potent than eqPO_3_H. The axial anomeric sulfonates **7b** and **8b** were also more potent N2 inhibitors relative to eqPO_3_H. In the case of CpNA inhibition, **7a** was 8.5-fold more potent than eqPO_3_H.

**Table 3:** *K*_*i*_ (inhibitory constant) and IC_50_ (50% inhibition constant) values for the inhibition of N1, N2, CpNA, and StrepNA.

	7a	7b	8b	eqPO_3_H
N1 *K*_*i*_	1.62 (1.08–2.27)	—	—	—
N1-His275Tyr *K*_*i*_	2.07 (1.63–2.62)	—	—	—
N2 *K*_*i*_^a^	2.47 (1.95–3.14)	33.5 (26.0–43.1)	88.1 (72.8–107)	103 (82.6–128)
N2 IC_50_	8.86 (6.99–11.3)	120 (93.0–155)	316 (260–384)	368 (296–458)
CpNA IC_50_	37.5 (31.2–45.0)	—	—	320 (221–460)
StrepNA IC_50_	3.74 (2.56–5.43)	—	—	—

All values are given in *μ* m units and 95% confidence intervals are listed inside parenthesis. ^a^Influenza N2 *K*_*i*_ values were estimated using the Cheng-Prusoff equation (*K*_*i*_ = IC_50_/(1 + [S]/*K*_*m*_))^23^ with the N2 *K*_*m*_ previously determined as 46.5 *μ* M ^3^. Dashes (−) indicate that inhibition was not determined.

**Figure 4:**
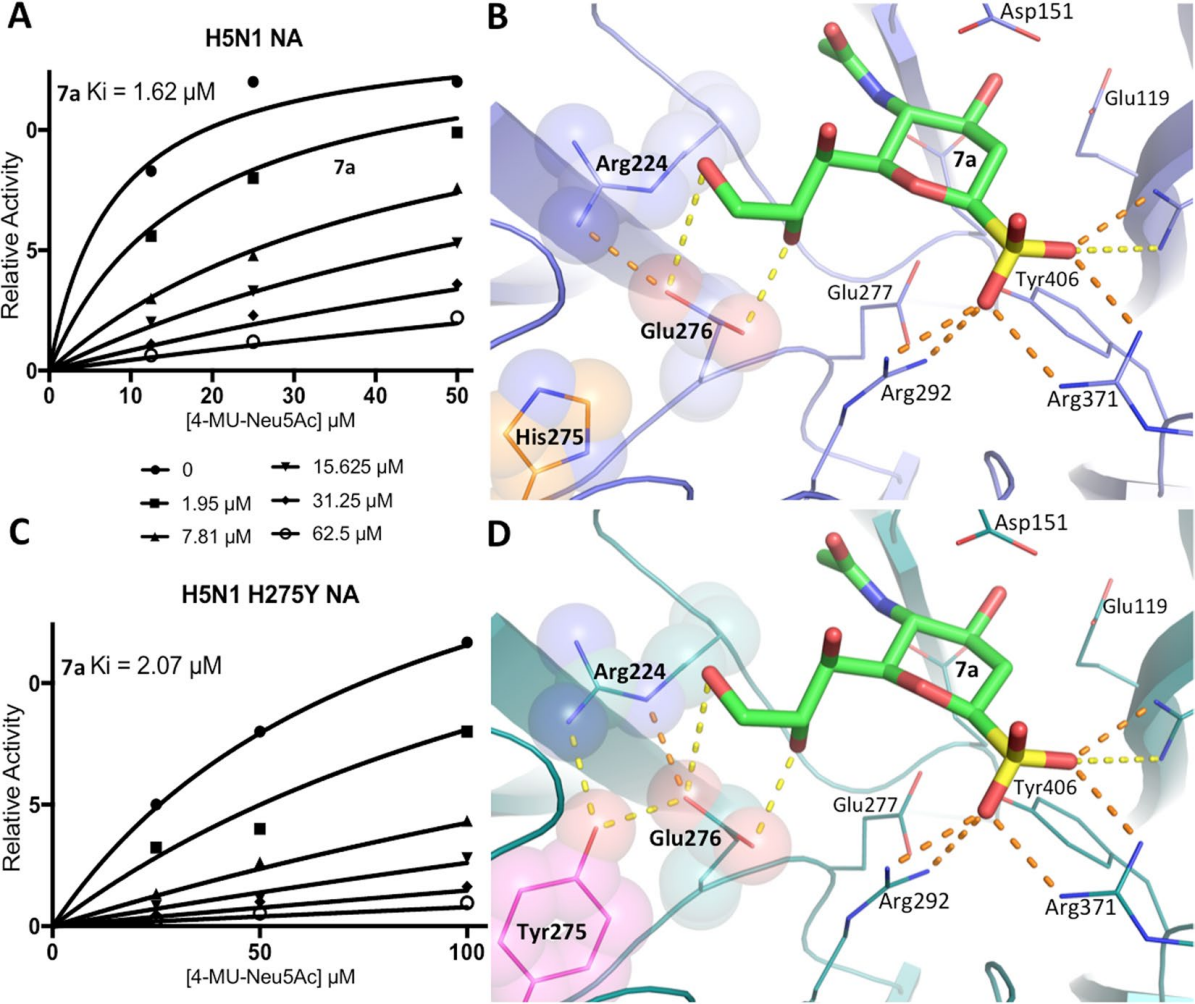
Inhibition of avian-origin H5N1 N1 by sulfo-sialic acid analogue **7a**. (**A**) **7a** inhibits wildtype N1 with an inhibitory constant (*K*_*i*_) of 1.62 μM. (**B**) Predicted binding of **7a** to the wildtype N1 active site. (**C**) **7a** inhibits His275Tyr oseltamivir-resistant N1 with a similar potency to that of wildtype N1. (**D**) Predicted binding of **7a** to the His275Tyr N1 active site shows that the **7a** glycerol side chain maintains the same interactions with the Glu276 side chain. Key residues relating to His275Tyr drug resistance are shown inside transparent spheres. Electrostatic interactions of the inhibitor sulfo group and glycerol moiety are indicated by dashed lines, with stronger ionic interactions colored orange. The binding analysis was performed with MOE based on the structure of A/Vietnam/1203/04 (H5N1) NA (PDB ID 3CKZ)^26^.

The sialic acid anomeric sulfo modification is advantageous in terms of drug resistance because of the essential role of the triarginyl binding site for natural NA substrate binding. Substitution of Arg118, Arg292 and Arg371 is known to result in impaired viral fitness due to decrease in NA activity and substrate affinity^24^. Although the Arg292Lys substitution is well documented, we showed that it results in an 88-fold *K*_*m*_ increase and a decrease in the enzymatic activity of H7N9 NA^25^. One of the most common forms of NA inhibitor resistance is caused by a His275Tyr substitution, which confers a high level of resistance to oseltamivir and moderate resistance to peramivir^24, 26^. Consistent with the results for A/RI/5+/1957(H2N2) NA, **7a** inhibits H5N1 N1 and its drug-resistant His275Tyr form at the same level (Table 3, Fig. 4), demonstrating the effectiveness of sulfo-sialic acid analogues against highly-prevalent drug-resistant NA and highly-pathogenic avian-origin NA.

Sialic acid anomeric phosphonic acid analogues were first reported by Walliman and Vasella in 1990^4^. The long delay in the synthesis of sulfo-sialic acid analogues might be explained by challenges with the current synthesis. Some difficulty includes the similar chromatographic mobility of the anomeric acetoxy and anomeric acetylthio intermediates, the precise conditions of the acetylthio oxidation, difficulty in sulfo protection, and high water solubility of the protected sulfo-sialic acid analogues.

The clinically used NA inhibitors zanamivir, oseltamivir, peramivir and laninamivir inhibit influenza NA at a low nanomolar level^1^. Despite having a lower potency, the sulfo-sialic acid analogue **7a** contains no other structural improvements and is still a more potent inhibitor of influenza NA than the oxocarbenium ion transition state analogue Neu4Ac2en (N2 IC_50_: 19 μm)^27^. Therefore, our sulfo modification in combination with other modifications may offer potential to produce sub-nanomolar level NA inhibitors. In conclusion, this report proves the concept that sulfo-sialic acid analogues are the most potent NA inhibitors with regards to anomeric functionality. Further development of novel sulfo-sialic acid based inhibitors with increased potency and efficacy is currently underway.

## Methods

### NMR Analysis

Compounds were analyzed using Varian 400 MHz and 600 MHz systems (now Agilent) at the Okayama University Collaboration Center, and also using a JEOL ECZ400 spectrometer at the Kobe University Department of Chemical Science and Engineering. Measurements were taken with **2-4** dissolved in CDCl_3_, and **5-8** dissolved in CD_3_OD. NMR chemical shifts (δ) are provided in parts per million (ppm).^1^H-NMR coupling constants (*J*) are listed in Hz. Residual peaks of chloroform (7.26 ppm) and methanol (3.31 ppm) were used as^1^H-NMR references. The residual peaks of chloroform (77.0 ppm) and methanol (49.0 ppm) were also used as^13^C-NMR references. For 2D experiments, the standard pulse sequences programmed into the Varian NMR systems were used. 2D-NOESY experiments were performed at room temperature with a spectral width of 9615.4 Hz (sw), an acquisition time of 0.15 seconds (at), 2844 data points (np), a filter bandwidth of 4000 Hz (fb), a relaxation delay of 2.000 seconds (d1), 4 transients (nt), 128 or 200 experiments (ni), a pulse width of 9.700 or 10.900 microseconds (pw) and a mixing time 0.500 seconds (mixN).

### High Resolution Mass Spectrometry (HRMS)

Compound identities were confirmed with HRMS, which was performed using an Agilent 6520 Accurate-Mass Q-TOF at the Okayama University Collaboration Center.

### Neuraminidase Inhibition

Inhibition of neuraminidase (NA) activity was quantified using a fluorogenic assay based upon the method by Potier *et al*.^22^ with modifications. 4-methylumbelliferyl-α-d-Neu5Ac (4-MU-Neu5Ac) substrate was utilized at a final concentration of 120 μm for inhibition of N2 and *Streptococcus* 6646K NA (Amsbio). For inhibition of *Clostridium perfringens* NanJ NA (Sigma) the 4-MU-Neu5Ac concentration was 60 μm when using **7a** and 120 μm when using eqPO_3_H. For IC_50_ determination, 5–8 inhibitor concentrations were used with 1–4 replicates for each condition. For N1 *K*_*i*_ determination, 5 inhibitor concentrations and 3 substrate concentrations were used. NA and inhibitor were pre-incubated for 20 minutes at room temperature before starting each assay with addition of 4-MU-Neu5Ac. Fluorescence of liberated 4-methylumbelliferyl (Ex. 355–360 nm, Em. 460 nm) was detected at room temperature using a Thermo Scientific Varioskan™ Flash or a Molecular Devices SpectraMax Paradigm microplate reader. IC_50_ values and confidence intervals were calculated by analyzing inhibition data using the ‘log(inhibitor) vs. response (three parameters)’ function of Prism 7 software. N1 *K*_*i*_ values were calculated using the Michaelis-Menten function of Prism 7, and N2 *K*_*i*_ values were calculated using the Cheng-Prusoff equation^23^. A/Anhui/1/2005 (H5N1) NAs were purchased from Sino Biological. Influenza A/RI/5+/1957(H2N2) NA was produced according to our previously reported methods^3^.

### Modeling Sulfo-Sialic Acid Analogue Binding

Sulfo-sialic acid analogue binding to NA active sites was modeled with Molecular Operating Environment (MOE) version 2016.0802 from Chemical Computing Group. The structure of **7a** was built in the A/RI/5+/1957(H2N2) N2 (PDB ID: 45H2) and A/Vietnam/1203/04 (H5N1) His275Tyr N1 (PDB ID: 3CKZ) active sites using the MOE Builder function. This was followed by running Structure Preparation, Protonate 3D, and Energy Minimization with the Amber10:EHT forcefield. Binding of **7a** to wildtype N1 was performed in the same manner as that of the N1-His275Tyr structure (PDB ID: 3CKZ) after substitution of Try275 with histidine using the MOE Protein Builder function.

#### Synthesis of 4-Acetamido-3,6,7,8-tetra-*O*-acetyl-1-acetylthio-1,2,4-trideoxy-d-*glycero*-d-*galacto*-octopyranose (3) and 4-Acetamido-6,7,8-tri-*O*-acetyl-1-acetylthio-2,3-didehydro-1,2,3,4-tetradeoxy-d-*glycero*-d-*galacto*-octopyranose (4)

The known compound 4-acetamido-1,3,6,7,8-penta-*O*-acetyl-2,4-dideoxy-d-*glycero*-d-*galacto*-octopyranose (**2**) was synthesized based upon the methods of Shie *et al*.^5^ with a 49% yield. **2** (0.4-1 g, 0.69-2 mmol) was dissolved in distilled 1,2-dichloroethane (1,2-DCE) or CH_3_CN dried with excess molecular sieves. After cooling, HSAc and Lewis acid (BF_3_·Et_2_O or TMSOTf) were carefully added to the solution according to Table 1. The reaction was kept under an atmosphere of N_2_ and the temperature was slowly raised to room temperature. After completion, the organic layer was combined with CHCl_3_ and washed with aqueous solutions of NaHCO_3_ and NaCl. The washed organic layer was dried over MgSO_4_, filtered through diatomaceous earth and evaporated under reduced pressure. Acetylthio reaction products **3** and **4** were isolated using normal phase column chromatography with EtOAc as the mobile phase. When using the polar conditions of Table 1 entry 1, 63 μmol **3a** was obtained from 426 μmol **2** (14% yield); when using the non-polar conditions of Table 1 entry 2, 766 μmol **3b** was obtained from 1.97 mmol **2** (39% yield); and when using the more non-polar conditions of Table 1 entry 3, 176 μmol **4b** was obtained from 673 μmol **2** (26% yield).

**3a** (α-Anomer): *Rf =* 0.4 (EtOAc); ^1^H-NMR (400 MHz, CDCl_3_): 1.89 (s, 3H, NH*Ac*), 2.0-2.13 (4s, 12H, 4 OAc), 2.34 (s, 3H, SAc), 3.77 (dd, 1H, *J*_5,4_ = 10.4, *J*_5,6_ = 2.4, H-5), 4.0 (m, 1H, H-4), 4.36 (dd, 1H, *J*_8,7_ = 2.5, *J*_8,8′_ = 12.5, H-8), 5.36 (dd, 1H, *J*_6,5_ = 2.4, *J*_6,7_ = 7.1, H-6); + ESI HRMS calcd for C_20_H_29_NNaO_11_S: 514.1359, found: *m/z* 514.1393 [M + Na]^+^.

**3b** (β-Anomer): *Rf = *0.4 (EtOAc); ^1^H-NMR (600 MHz, CDCl_3_): 1.89 (1s, 3H, NH*Ac*), 1.98–2.12 (4s, 12H, 4 OAc), 2.16–2.19 (ddd, 1H, H-2ax), 2.30–2.36 (m, 1H, H-2eq), 2.37 (s, 3H, SAc), 3.91 (dd, 1H, *J*_5,4_ = 10.4, *J*_5,6_ = 1.9, H-5), 4.00–4.05 (m, 2H, H-4, H-8) 4.27 (dd, 1 H, *J*_8′,7_ = 2.9, *J*_8′,8_ = 12.6, H-8′), 5.00 (ddd, 1H, *J*_3,2eq_ = 4.7, *J* = 10.3, *J* = 11.7, H-3), 5.12 (ddd, 1H, *J*_7,6_ = 7.2, *J*_7,8_ = 6.3, *J*_7,8′_ = 2.9, H-7), 5.31 (dd, 1H, *J*_6,5_ = 1.9, *J*_6,7_ = 7.2, H-6), 5.41 (d, 1H, *J*_NH,4_ = 10, NH), 6.14 (d, 1H, *J*_1,2ax_ = 4.7, H-1); ^13^C-NMR (150 MHz, CD_3_OD): 20.67, 20.69, 20.8, 20.9, 21.05, 23.15 (C=O*C*H_3_), 36.1 (C-2), 50.1, 62.1, 67.3, 69.6, 69.9, 72.5 (C-3–C-8), 79.0 (C-1), 169.4, 170.0, 170.13, 170.16, 170.5, 171.0 (C=OCH_3_); + ESI HRMS calcd for C_20_H_29_NNaO_11_S: 514.1359, found: *m/z* 514.1393 [M + Na]^+^.

**4b** (β-Anomer): *Rf = *0.4 (EtOAc):^1^H-NMR (600 MHz, CDCl_3_): 1.98–2.12 (4s, 12H, 4 C=O*C*H_3_), 2.35 (s, 3H, SAc) 3.83 (dd, 1H, *J*_5,4_ = 10, *J*_5,6_ = 1.8, H-5), 4.11 (dd, 1H, *J*_8,7_ = 5.7, *J*_8,8′_ = 12.5, H-8), 4.30 (dd, 1H, *J*_8′,7_ = 2.6, *J*_8′,8_ = 12.6, H-8′), 4.50 (t, 1H, *J*_4,3_ = 9.8, *J*_4,5 = _9.8, H-4), 5.21 (ddd, 1H, *J*_7,6_ = 7.8, *J*_7,8_ = 5.6, *J*_7,8′_ = 2.6, H-7), 5.31 (dd, 1 H, *J*_6,5_ = 1.8, *J*_6,7_ = 7.8, H-6), 5.53 (d, 1H, *J*_NH,4_ = 9.4, NH) 5.80 (s, 2H, H-2, H-3) 6.27 (s, 1H, H-1); ^13^C-NMR (150 MHz, CD_3_OD): 20.70, 20.73, 20.95, 21.1, 23.4 (5 C=O*C*H_3_), 43.6, 62.1, 67.4, 69.3, 70.1 (C-4–C-8), 78.7, (C-1), 125.7, 131.1 (C-2, C-3), 169.6, 169.8, 170.2, 170.53, 170.58 (5 C=OCH_3_); + ESI HRMS calcd for C_18_H_25_NNaO_9_S: 454.1148, found: *m/z* 454.1177 [M + Na]^+^.

#### Synthesis of 4-Acetamido-3,6,7,8-tetra-*O*-acetyl-1-sulfo-1,2,4-trideoxy-d-*glycero*-d-*galacto*-octopyranose (**5**)

**3** (427 mg, 869 mmol, **3a**:**3b** = 2:7) and **4b** (307 mg, 711 mmol) were dissolved in AcOH. NH_4_OAc (1.0 g, 13 mmol, 8.2 eq.) and Oxone (3.67 g, 11.9 mmol, 7.5 eq.) were mixed in to form a slurry. After 21 hours, MeOH was added to the reaction mixture, which was then filtered through a fritted funnel. The crude mixture was evaporated under reduced pressure, followed by purification of **5a** and **5b** (525 mg, **5a**:**5b** = 2:7, quantitative yield) with normal phase chromatography using a gradient of EtOAc and EtOH.

**5a** (α-Anomer): *Rf* = 0.39 (EtOAc:EtOH = 2:1); ^1^H-NMR (400 MHz, CD_3_OD): 1.8–2.0 (m, 1H, H-2ax) 1.85 (s, 3H, NH*Ac*), 1.97–2.08 (4s, 12H, OAc), 2.49 (ddd, 1H, *J*_2eq,1_ = 2.1, *J*_2eq,2ax_ = 12.6, *J*_2eq,3_ = 5.1, H-2eq), 3.81 (dd, 1H, *J*_5,4_ = 10.4, *J*_5,6_ = 2.3, H-5), 3.97 (ddd, 1H, *J*_4,3_ = 10.4, *J*_4,5_ = 10.4, *J*_4,NH_ = 10.4, H-4), 4.21 (dd, 1H, *J*_8,7_ = 6.3, *J*_8,8′_ = 12.3, H-8), 4.26 (dd, 1H, *J*_1,2ax_ = 11.8, *J*_1,2eq_ = 2.1, H-1), 4.53 (dd, 1H, *J*_8′,7_ = 2.8, *J*_8′,8_ = 12.4, H-8′), 5.00 (ddd, 1H, *J*_3,2ax_ = 11.3, *J*_3,2eq_ = 5.1, *J*_3,4_ = 10.2, H-3), 5.30 (ddd, 1H, *J*_7,6_ = 6.2, *J*_7,8_ = 6.2, *J*_7,8′_ = 2.7, H-7), 5.39 (m, 1H, H-6); ^13^C-NMR (150 MHz, CD_3_OD): 20.69, 20.79, 20.82, 20.82, 22.7 (5 C=O*C*H_3_), 33.6 (C-2), 50.5, 63.2, 69.4, 72.6, 73.2, 78.6 (C-3–C-8), 87.3 (C-1), 171.7, 171.8, 172.0, 172.5, 173.4 (5 C=OCH_3_); -ESI HRMS calcd for C_18_H_26_NO_13_S^−^: 496.1130, found: *m/z* 496.1142 [M − H]^−^.

**5b** (β-Anomer): *Rf* = 0.4 (EtOAc:EtOH = 2:1);^1^H-NMR (600 MHz, CD_3_OD): 1.84 (s, 3H, NH*Ac*) 1.9–2.1 (m, 1H, H-2ax), 1.97, 2.00, 2.07, 2.08 (4s, 12H, OAc), 2.68 (ddd, 1H, *J*_2eq,1_ = 1.0, *J*_2eq,2ax_ = 13.9, *J*_2eq,3_ = 5.4, H-2eq), 3.96 (t, 1H, *J*_4,3_ = 10.3, *J*_4,5_ = 10.3, H-4), 4.12 (dd, 1H, *J*_8,7_ = 5.3, *J*_8,8′_ = 12.6, H-8), 4.42 (dd, 1H, *J*_8′,7_ = 2.9, *J*_8′,8_ = 12.3, H-8′), 4.66 (d, 1H, *J*_1,2ax_ = 6.7, H-1), 4.77 (dd, 1H, *J*_5,4_ = 10.4, *J*_5,6_ = 2.2, H-5), 5.21 (ddd, 1H, *J*_7,6_ = 7.8, *J*_7,8_ = 5.1, *J*_7,8′_ = 2.6, H-7), 5.39 (dd, 1H, *J*_6,5_ = 2.2, *J*_6,7_ = 7.8, H-6), 5.58 (ddd, 1H, *J*_3,2ax_ = 10.5, *J*_3,2eq_ = 5.4, *J*_4,5_ = 10.5, H-3); ^13^C-NMR (150 MHz, CD_3_OD): 20.7, 20.8, 20.9, 21.3, 22.6 (5 C=O*C*H_3_), 31.5 (C-2), 50.7, 63.1, 69.1, 71.0, 71.0, 73.0 (C-3–C-8), 85.8 (C-1), 171.7, 172.0, 172.2, 172.6, 173.5 (5 C=OCH_3_); -ESI HRMS calcd. for C_18_H_26_NO_13_S^−^: 496.1130, found: *m/z* 496.1142 [M − H]^−^.

#### Synthesis of 4-Acetamido-6,7,8-tri-*O*-acetyl-1-sufo-2,3-didehydro-1,2,3,4-tetradeoxy-d-*glycero*-d-*galacto*-octopyranose (**6**)

**4b** (76 mg, 176 μmol) was dissolved in AcOH (3 mL). KOAc (Wako, 190 mg, 1.94 mmol, 9.85 eq.) and Oxone (378 mg, 1.23 mmol, 6.24 eq.) were mixed in to form a slurry. After 4.66 hours MeOH was added to the reaction mixture, which was then filtered through a fritted funnel. The crude mixture was evaporated under reduced pressure, followed by purification of **6b** (10.5 μmol, 6% yield) using normal phase column chromatography.

**6b** (β-Anomer): *Rf* = 0.39 (EtOAc:EtOH = 2:1);^1^H-NMR (600 MHz, CD_3_OD): 1.92, 2.01, 2.08 (3s, 12H, 4 Ac), 4.21 (dd, 1H, *J*_8,7_ = 5.5, *J*_8,8′_ = 12.4, H-8), 4.52 (m, 2H, H-4, H-8′), 4.60 (m, 1H, H-5), 4.91 (m, 1H, H-1), 5.28 (ddd, 1H, *J*_7,6_ = 7.4, *J*_7,8_ = 5.3, *J*_7,8′_ = 2.6, H-7), 5.40 (dd, 1H, *J*_6,5_ = 2.1, *J*_6,7_ = 7.1, H-6), 5.83 (dt, 1H, *J*_3,1_ = 2.1, *J*_3,2_ = 10.2, *J*_3,4_ = 2.1, H-3), 6.11 (dt, 1H, *J*_2,1_ = 2.9, *J*_2,3_ = 10.1, *J*_2,4_ = 2.9, H-2);^13^C-NMR (150 MHz, CD_3_OD): 20.7, 21.3, 22.6, 23.7 (4 C=O*C*H_3_), 43.9, 63.1, 69.2, 71.5, 71.7 (C-4–C-8), 86.1 (C-1), 124.8, 131.9 (C-2, C-3), 171.6, 172.2, 172.6, 173.2 (4 C=OCH_3_); -ESI HRMS calcd for C_16_H_22_NO_11_S^−^: 436.0919, found: *m/z* 436.0929 [M − H]^−^.

#### Preparation of 4-Acetamido-1-sulfo-1,2,4-trideoxy-d-*glycero*-d-*galacto*-octopyranose (**7**) and 4-Acetamido-1-sufo-2,3-didehydro-1,2,3,4-tetradeoxy-d-*glycero*-d-*galacto*-octopyranose (**8**)

To prepare active sulfo-sialic acid analogues, **5a** (10 mg), **5b** (10 mg), and **6b** (4 mg) were treated with a solution of 0.1 m NaOH in MeOH. After deprotection reached completion, the pH was neutralized with AcOH and solvent was evaporated under reduced pressure resulting in quantitative final product yields. **7a**, **7b** and **8b** were purified to homogeneity using an Inertsil^®^ ODS-3 semi-preparative HPLC column (GL Sciences) with a mobile phase of 10–15% MeOH supplemented with NH_4_OAc.

**7a** (α-Anomer): *Rf* = 0.6 (acetone:butan-1-ol:H_2_O = 70:27:3); ^1^H-NMR (600 MHz, CD_3_OD): 1.79 (ddd, 1H, *J*_2ax,1_ = 12, *J*_2ax,2eq_ = 12, *J*_2ax,3_ = 12, H-2ax), 2.00 (s, 3H, Ac), 2.45 (ddd, 1H, *J*_2eq,1_ = 1, *J*_2eq,2ax_ = 14, *J*_2eq,3_ = 5.5, H-2eq), 3.47 (dd, 1H, *J*_6,5_ = 0.9, *J*_6,7_ = 8.8, H-6), 3.62–3.65 (m, 2H, H-5, H-8), 3.74–3.89 (m, 4H, H-3, H-4, H-7, H-8′), 4.28 (dd, 1H, *J*_1,2ax_ = 11.7, *J*_1,2eq_ = 2.1, H-1); ^13^C-NMR (150 MHz, CD_3_OD): 22.8 (C=O*C*H_3_), 36.4 (C-2), 53.7, 64.9, 70.2, 70.8, 71.4, 78.6 (C-3–C-8), 87.0 (C-1), 174.8 (C=OCH_3_); -ESI HRMS calcd for C_10_H_18_NO_9_S^−^: 328.0708, found: *m/z* 328.0727 [M − H]^−^.

**7b** (β-Anomer): *Rf* = 0.65 (acetone:butan-1-ol:H_2_O = 70:27:3); ^1^H-NMR (600 MHz, CD_3_OD): 1.92 (ddd, 1H, *J*_2ax,1_ = 7, *J*_2ax,2eq_ = 14, *J*_2ax,3_ = 10.9, H-2ax), 2.01 (s, 3H, Ac), 2.62 (ddd, 1H, *J*_2eq,1_ = 1, *J*_2eq,2ax_ = 14, *J*_2eq,3_ = 5.5, H-2eq), 3.44 (dd, 1H, *J*_6,5_ = 1.2, *J*_6,7_ = 9.1, H-6), 3.57–3.60 (m, 1H, H-8), 3.75 (t, 1H, *J*_4,3_ = 10.1, *J*_4,5_ = 10.1, H-4), 3.79–3.84 (m, 2H, H-7, H-8′), 4.25 (dd, 1H, *J*_5,4_ = 10.6, *J*_5,6_ = 1.5, H-5), 4.40 (ddd, 1H, *J*_3,2ax_ = 10.3, *J*_3,2eq_ = 5.4, *J*_3,4_ = 10.3, H-3), 4.70 (d, 1H, *J*_1,2ax_ = 6.7, H-1); ^13^C-NMR (150 MHz, CD_3_OD): 22.6 (C=O*C*H_3_), 34.3 (C-2), 54.3, 64.6, 67.2, 70.2, 72.4, 74.3 (C-3–C-8) 86.7 (C-1), 175.3 (C=OCH_3_); -ESI HRMS calcd for C_10_H_18_NO_9_S^−^: 328.0708, found: *m/z* 328.0727 [M − H]^−^.

**8b** (β-Anomer): *Rf* = 0.6 (acetone:butan-1-ol:H_2_O = 70:27:3); ^1^H-NMR (600 MHz, CD_3_OD): 1.98 (s, 3H, Ac), 3.50 (dd, 1H, *J*_6,5_ = 1.5, *J*_6,7_ = 9.1, H-6), 3.62 (dd, 1 H, *J*_8,7_ = 6.2, *J*_8,8′_ = 11.4, H-8), 3.83 (dd, 1H, *J*_8′,7_ = 2.9, *J*_8′,8_ = 11.4, H-8′), 3.92 (ddd, 1H, *J*_7,6_ = 9.1, *J*_7,8_ = 6.2, *J*_7,8′_ = 2.9, H-7), 4.35 (dd, 1H, *J*_5,4_ = 9.5, *J*_5,6_ = 1.6, H-5), 4.68 (dddd, 1H, *J*_4,1_ = 2.3, *J*_4,2_ = 2.3, *J*_4,3_ = 2.3, *J*_4,5_ = 9.5, H-4), 4.96 (ddd, 1H, *J*_1,2_ = 2.6, *J*_1,3_ = 2.6, *J*_1,4_ = 2.6, H-1), 5.93 (dt, 1H, *J*_3,1_ = 2.1, *J*_3,2_ = 10.3, *J*_3,4_ = 2.1, H-3), 6.13 (dt, 1H, *J*_2,1_ = 2.8, *J*_2,3_ = 10.3, *J*_2,4_ = 2.8, H-2); ^13^C-NMR (150 MHz, CD_3_OD): 22.6 (C=O*C*H_3_), 44.6, 64.9, 70.1, 72.4, 73.0 (C-4–C-8), 86.3 (C-1), 124.9, 132.0 (C-2, C-3), 173.9 (C=OCH_3_); -ESI HRMS calcd for C_10_H_16_NO_8_S^−^: 310.0602, found: *m/z* 310.0578 [M − H]^−^.

### Data Availability

The datasets generated and analyzed during the current study are available from the corresponding authors upon reasonable request.
